# Air-Coupled Ultrasonic Optical Coherence Elastography Reveals Protocol-Dependent Corneal Stiffening in Epi-On and Epi-Off Riboflavin Crosslinking

**DOI:** 10.1167/tvst.15.7.14

**Published:** 2026-07-10

**Authors:** Fernando Zvietcovich, Alejandra Varea-Bejar, Vlada Pleshcheva, Andrea Curatolo, Judith S. Birkenfeld, Susana Marcos

**Affiliations:** 1 Department of Engineering, Pontificia Universidad Catolica del Peru, Lima, Peru; 2 “Daza de Valdes” Institute of Optics, Spanish National Research Council, Madrid, Spain; 3 Department of Physics, Politecnico di Milano, Milan, Italy; 4 Center for Visual Science, The Institute of Optics, Flaum Eye Institute, University of Rochester, NY, USA

**Keywords:** corneal crosslinking, accelerated protocols, corneal biomechanics, optical coherence elastography, wave propagation

## Abstract

**Purpose:**

To evaluate the biomechanical impact of corneal crosslinking (CXL) when combining three different ultraviolet A (UV-A) riboflavin (RB) photosensitizers and protocols (Dresden protocol [DP] and accelerated protocol [AP]) using air-coupled ultrasonic optical coherence elastography in ex vivo rabbit corneas.

**Methods:**

An air-coupled ultrasound excitation optical coherence elastography system was used to excite the corneal apex and generate Lamb wave propagation along 16 corneal cross-sectional meridians. Measurements were conducted on ex vivo rabbit eyes (*n* = 45) during three treatment phases: Virgin, after 30 minutes of RB soaking, and after UV-A irradiation. The protocols used include the DP (3 mW/cm^2^, 30 minutes) and two accelerated protocols (AP-1: 9 mW/cm^2^, 10 minutes; and AP-2: 30 mW/cm^2^, 3 minutes). Each protocol was tested with three photosensitizers: epithelium-on (TE), and epithelium-off with hyperosmolar (M) and hypo-osmolar solutions (D). Lamb wave speed and average corneal thickness were calculated for each meridian to estimate meridian-dependent corneal shear modulus. Differences were statistically analyzed using linear mixed-effects regression.

**Results:**

The DP-D produced the strongest significant corneal stiffness increase (480.5 kPa; *P* < 0.001) during the UV irradiation phase, and the most pronounced corneal thinning (186 µm; *P* < 0.001). DP-TE achieved the next strongest stiffening (309.5 kPa) during UV irradiation, with no significant thickness change. All other protocol–photosensitizer combinations did not achieve significant corneal stiffening except for AP-1-D and AP-2-D during the RB soaking phase.

**Conclusions:**

Accelerated protocols did not produce significant corneal stiffening for any RB photosensitizer except when using dextran-based D. DP-D and DP-TE produced the greatest balance between shear modulus increase and corneal thickness decrease, suggesting DP-TE as a potential compromise between biomechanical impact, corneal integrity, and faster postoperative recovery.

**Translational Relevance:**

This work quantified the impact of clinically used crosslinking photosensitizers and protocols on corneal stiffening using an air-coupled ultrasound excitation optical coherence elastography, which holds promise for clinical assessments in patients.

## Introduction

Corneal crosslinking (CXL) has emerged as a critical therapeutic intervention in ophthalmology, particularly for treating corneal ectasia and keratoconus.^1^ By inducing photochemical CXL of stromal collagen, CXL enhances corneal rigidity and stabilizes its shape, preventing the progression of biomechanical deterioration associated with these pathologies.^2^ The original Dresden protocol (DP), which involves ultraviolet A (UV-A) irradiation combined with riboflavin (RB) as a photosensitizer, remains the gold standard for CXL treatments.^3^ However, there are concerns regarding the duration of the procedure (1 hour) and the induced corneal thinning that may induce patient discomfort and risk of corneal damage. Alternatives to overcome this problem include the use of different irradiation wavelengths and photosensitizers (e.g., green light and Rose Bengal),^4^ or the same UV-A with different irradiation regimes (accelerated protocols [APs]) and osmolarity of the photosensitizer solution. UV-A–accelerated CXL protocols and new RB preparations seek to optimize treatment by reducing the total procedure time (3–10 minutes), avoiding corneal thickness reduction while preserving biomechanical efficacy.^5^ Besides, removing the epithelium in corneal CXL enhances RB penetration and UV-A efficacy, increasing treatment effectiveness; however, it also increases the risk of postoperative pain, infection, and delayed epithelial healing. For this reason, protocols aimed at preserving the epithelium have been proposed.^6^

The biomechanical effects of CXL are primarily characterized by increased corneal stiffness and enhanced resistance to enzymatic degradation. CXL induces structural alterations within the collagen fibril network, strengthening interfibrillar bonds and thereby improving the overall mechanical integrity of the cornea.^2^^,^^5^^,^^7^ Biomechanically, the corneal stroma can be approximated as a transversely isotropic material,^8^^,^^9^ whereby CXL-induced stiffening manifests as an increase in both the out-of-plane shear modulus (relative to corneal surface), and the in-plane Young's modulus (parallel to corneal surface).^8^ While the conventional DP produces a marked and uniform stiffening effect,^10^^–^^13^ APs tend to result in a more superficial CXL, raising concerns about their capacity to achieve equivalent corneal biomechanical reinforcement.^5^^,^^14^^–^^18^

To address the limitations of the DP, APs were developed to shorten treatment time by increasing the UV-A irradiance while maintaining, a constant total energy dose of 5.4 J/cm² across all cases. Although standard CXL uses an irradiance of 3 mW/cm² for 30 minutes,^3^ APs use higher intensities (9–30 mW/cm²) with shorter exposure times (3–10 minutes).^5^ These accelerated variations are based on the Bunsen–Roscoe law of reciprocity, which states that the photochemical effect of light exposure is directly proportional to the total energy received, independent of duration.^19^ However, this reciprocity does not hold linearly in corneal CXL, as oxygen diffusion becomes limited at higher irradiances, resulting in a reduced CXL effect.^20^^,^^21^

Parallel to procedural advances, considerable effort has been directed toward developing novel RB formulations to modulate corneal hydration and enhance stromal penetration during CXL. Traditional dextran-based solutions, such as those used in the original DP, facilitate controlled corneal hydration to optimize CXL depth, but they are associated with significant intraoperative corneal thinning.^5^ In contrast, emerging dextran-free formulations have been designed to enhance RB diffusion into the stroma while minimizing dehydration, thereby supporting the feasibility of transepithelial (epi-on) CXL approaches.^22^^–^^24^ However, the interaction between these formulations and specific CXL protocols may substantially influence treatment efficacy, with some studies indicating reduced biomechanical reinforcement relative to conventional epithelium-off (epi-off) protocols.^14^^–^^17^^,^^25^ Therefore, the quantitative evaluation of stromal stiffening across novel RB formulations and protocols, benchmarked against the Dresden standard, is critical for elucidating the trade-off between biomechanical strengthening and preservation of corneal integrity—particularly under conditions approximating clinical practice.

This study presents a comparative analysis of the biomechanical impact of UV-A RB-mediated corneal CXL in ex vivo rabbit corneas maintained at a physiological intraocular pressure (IOP) of 15 mm Hg. Three irradiation protocols were evaluated: the standard DP (30 minutes, 3 mW/cm²) and two APs (10 minutes of irradiation, 9 mW/cm^2^; and 3 minutes of irradiation, 30 mW/cm^2^), each combined with one of three RB photosensitizers: epithelium-off using either a dextran-based or dextran-free formulation, and epithelium-on (TE) using a transepithelial dextran-free solution. Biomechanical parameters were assessed at three stages: baseline (Virgin), post-RB soaking (Post-RB), and post-irradiation (Post-CXL). Using air-coupled ultrasound excitation optical coherence elastography (ACUS-OCE),^26^^–^^28^ we quantified changes in the corneal shear modulus and thickness changes across protocol–photosensitizer combinations. These measurements aimed to elucidate the balance between mechanical reinforcement and structural preservation, informing optimization strategies for future clinical translation of CXL techniques.

## Material and Methods

### Sample Preparation

**Figure 1.**
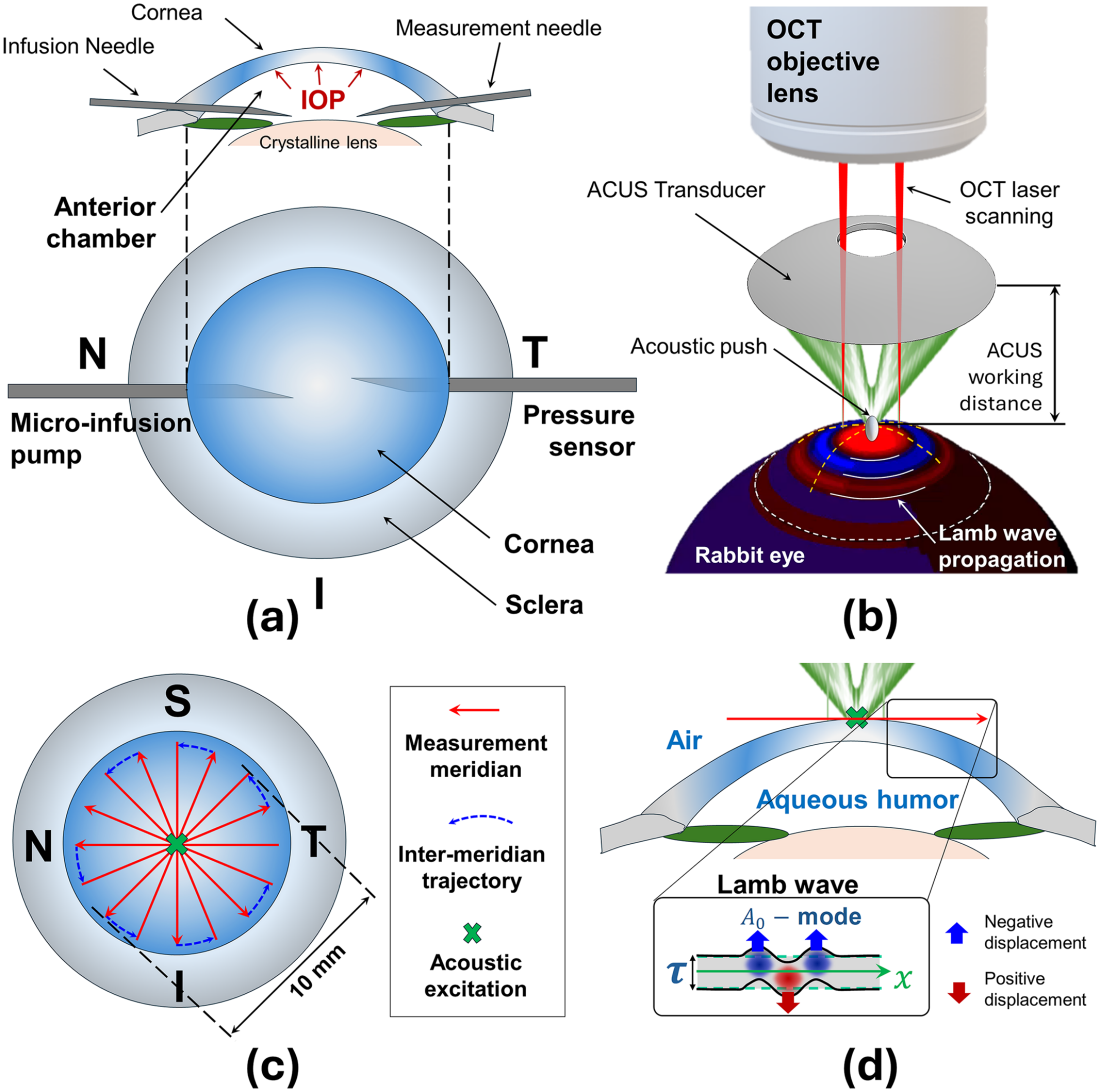
(**a**) Pressurized rabbit ex vivo eye globe using a closed loop IOP control system set to 15.0 ± 0.2 mm Hg. (**b**) ACUS-OCE system setup composed of a PhS-SS-OCT system and a 500-kHz ACUS transducer, confocal and coaligned with the OCT sample arm. The acoustic excitation is focused on the corneal apex. (**c**) Acquisition of 8 OCT meridians distributed along 360° (steps of 22.5°), forming 16 semimeridians centered at the corneal apex. (**d**) Outward antisymmetric 0-mode Lamb wave propagation along a single corneal meridian after excitation. I, inferior; N, nasal; S, superior; T, temporal.

Forty-five freshly enucleated rabbit eyes from adult New Zealand white rabbits (2–3 kg) were obtained (Facultad de Veterinaria, Complutense University of Madrid, Madrid, Spain) and used for experimental procedure within 24 hours post mortem. The anatomical orientation was identified during the eye enucleation process and marked for subsequent location into the eye holder. Before measurements, muscles and conjunctival tissue were removed from the sclera. After preparation, the intact eye was placed into a customized holder that allows for ocular canulation using two 25G needles that penetrated into the anterior chamber through the limbus along nasal and temporal regions (Fig. 1a). The eyes were systematically aligned so that their anatomical superior–inferior meridian coincides with the vertical scanning axis in the holder. One needle was connected to a pressure sensor, and the other was connected to a controlled fluid pump system. Both the pressure sensor and the microinfusion pump were fully controlled by the computer through a closed loop system that ensured constant IOP of 15 ± 0.2 mm Hg during all measurements.^26^

### Corneal CXL Treatments and Protocols

Biomechanical outcomes were investigated for three CXL protocols (DP and two APs, AP-1 and AP-2) each combined with three RB photosensitizers: (D) a dextran-based, epi-off solution (0.1% RB, 20% dextran: Dresden solution, isotonic; MedioCross Glaukos Corp, Aliso Viejo, CA); (M) a dextran-free epithelium-off solution (epi-off, 0.1% RB, 1.1% hydroxypropyl methylcellulose [HPMC], isotonic); and (TE) transepithelial dextran-free solution for TE application (epi-on, 0.25% RB, 1.2% HPMC, 0.01% benzalkonium chloride [BKC]), as summarized in Table 1. The protocols used in this study included the DP and two accelerated (AP-1, AP-2) CXL protocols, maintaining a constant total energy dose of 5.4 J/cm² in all cases, as described in Table 2. A total of 45 eyes were distributed in different CXL sessions obtained from the combination of all protocols (DP, AP-1, and AP-2), and all RB photosensitizers (D, M, and TE), resulting in 5 eyes in each of the 9 [protocol]–[photosensitizer] cases.

**Table 1.** Summary of Corneal CXL Photosensitizers

Photosensitizers	Transepithelial	Dextran	RB	Protocol Used
D	No	20%	0.1%	DP, AP-1, AP-2
M	No	0%	0.1%	DP, AP-1, AP-2
TE	Yes	0%	0.25%	DP, AP-1, AP-2

**Table 2.** Summary of Corneal CXL Protocols

Protocol	RB Soaking, Minutes	UV-A Irradiation, Minutes	Irradiance, mW/cm²	RB Application During UV-A	Photosensitizers Used
DP	30	30	3	Every 5 min	D, M, TE
AP-1	30	10	9	Every 3 min	D, M, TE
AP-2	30	3	30	Every 1 min	D, M, TE

UV-A irradiation was delivered using a commercial LED (365 nm central wavelength, 9 nm full-width half-maximum bandwidth) lamp mounted with an aspheric collimation lens (EFL = 40 mm, AR-coated) compatible with Leica microscopes (M365LP1-C2, Thorlabs, Newton, NJ) yielding a 37-mm beam diameter and a total power of 435 mW. A custom-designed three-dimensionally printed mask was positioned anterior to the light source to confine the effective irradiation area to a 15-mm diameter circle and exposure of the limbus. The lamp was operated in a continuous-wave mode and calibrated with a UV radiometer (UVA-365SD, Lutron Electronic, Coopersburg, PA) to provide irradiances of 3 mW/cm^2^, 9 mW/cm^2^, and 30 mW/cm^2^, as specified by each CXL protocol.

### ACUS-OCE Setup

Biomechanical measurements of the corneas were obtained using a phase-sensitive swept-source optical coherence tomography (PhS-SS-OCT) system, coaligned in a confocal configuration with a custom-built air-coupled ultrasonic (ACUS) piezoelectric transducer for tissue excitation at the corneal apex,^29^^–^^31^ as shown in Figure 1b. The PhS-SS-OCT is a custom-built system described in detail in previous publications^32^^–^^34^ featuring a swept source (VCSEL, SL132120, Thorlabs) centered at a 1300-nm wavelength, with a spectral bandwidth of 50 nm, and an A-line scan rate of 200 kHz. The full-width half-maximum axial resolution is 16 µm in air, and a lateral resolution (sample arm) of 40 µm at the focal plane, and a 5.15-mm imaging depth of field in tissue.^32^ Tissue nanometer displacement was obtained in the sample over time in response to a stimulation using the phase information of the OCT spectrum through the phase-sensitive mode.^35^ The system allows multidirectional scanning via high-speed galvanometric scanning mirrors (Saturn 1B, Scanner MAX, Pangolin, New York, NY). In this study we acquired 8 corneal cross-sectional meridians (10 mm length) equally distributed over 360° (steps of 22.5°), forming 16 semimeridians centered at the corneal apex, as shown in Figure 1c.

The ultrasonic transducer was driven by a three-cycle tone burst, consisting of a 2.5-kHz train of square pulses modulating a 500-kHz carrier signal that was preamplified using a radiofrequency amplifier (100A250A, Amplifier Research, Souderton, PA). The excitation generated a radially symmetric acoustic radiation force at the corneal apex, producing a localized push with a lateral extent of 0.6 mm. The resulting deformation (∼1 µm) in the cornea initiated the outward propagation of a cylindrical Lamb wave, specifically the antisymmetric zero-order mode (A0), as illustrated in Figure 1d. Lamb wave propagation, along with structural imaging of the cornea, was acquired along each semimeridian using the PhS-SS-OCT system. The 15-mm central aperture of the ACUS transducer permitted unobstructed passage of the OCT beam, enabling co-localized optical access to the corneal sample.

### OCE Measurements and Processing

The M-B scanning protocol^35^ was used to capture wave propagation along eight cross-meridians of the cornea. At each lateral location, the ACUS was triggered and an M-mode frame consisting of 450 A-lines over time (temporal sampling interval Δ*t* = 5 µs) was acquired. This M-mode acquisition was repeated for each of the 100 spatial positions forming a 10-mm a cross-meridian (spatial resolution = 0.1 mm) and subsequently repeated for all eight cross-meridians. A 250-µs interval was implemented between consecutive steps to allow for stable lateral and angular transitions across all scanning positions (Fig. 1c). The total acquisition time was 2 seconds, including the transition periods allocated to the galvanometric scanning system to move smoothly between meridians, thereby minimizing mechanical oscillations and preventing mirror-induced vibrations.

Each cornea was measured using the ACUS-OCE system at three time points along the CXL treatment, also called treatment phases: (1) Virgin (referring to untreated epi-off cornea for D and M; and epi-on cornea for TE); (2) Post-RB (after 30 minutes of RB soaking); and (3) Post-CXL (after the UV irradiation). Drops of balanced salt solution were poured over the corneas to hydrate them before each OCE measurement.

**Figure 2.**
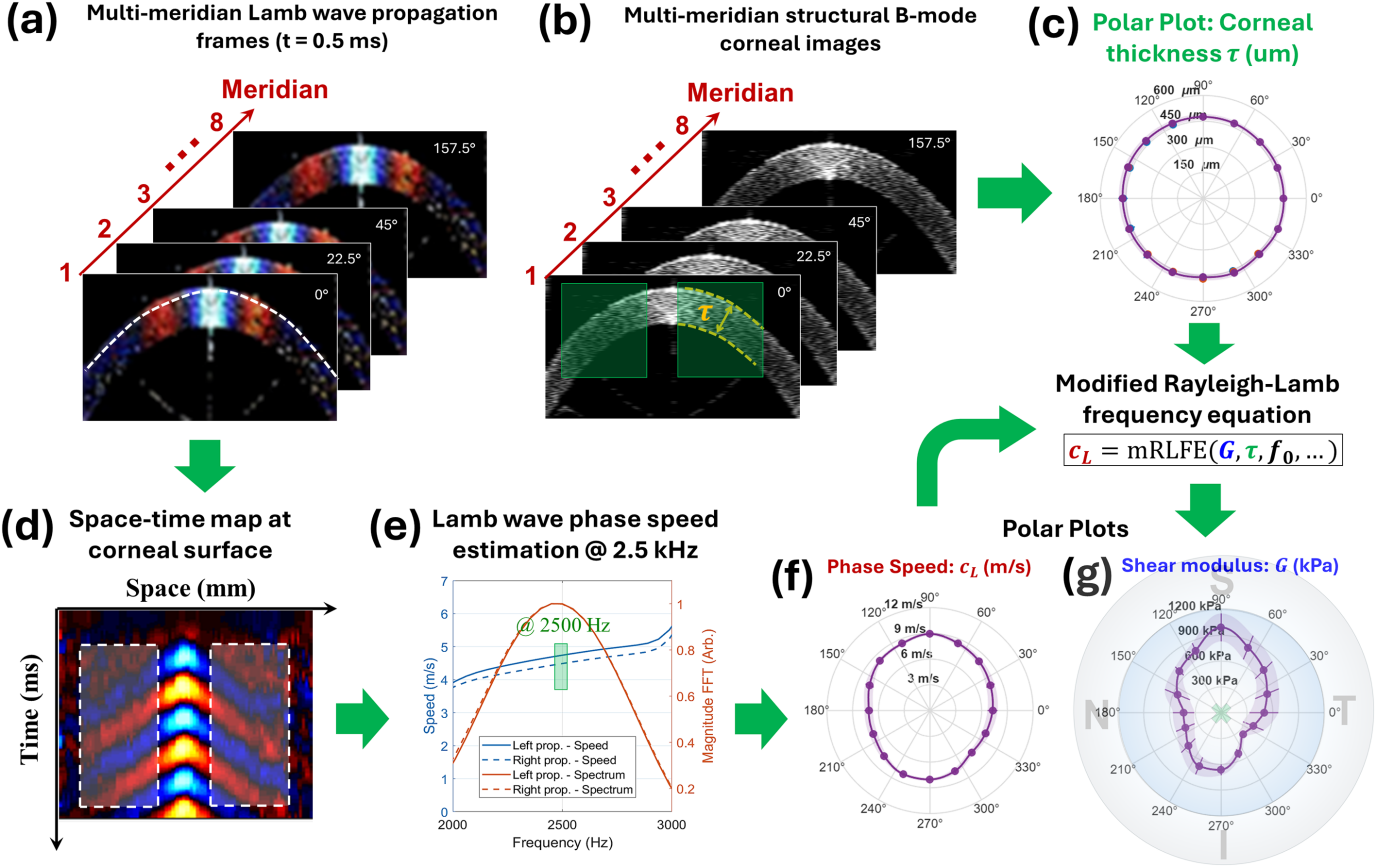
OCE processing approach. (**a**) Motion (particle velocity) wave propagation videos of each meridian. A representative frame at *t*_0_ = 0.5 ms is shown for each video. (**b**) Structural B-mode OCT images of the cornea of each meridian. We defined a left- and right-propagation spatial regions (4 mm × 4 mm size) in each B-mode image as shown om the *green rectangular region* of (**b**). Average corneal thickness polar plot (in µm) extracted from the left- and right-propagation spatial regions of (**b**) using thresholding techniques. (**d**) Space–time motion maps depicting wave propagation extracted form (**a**) at the corneal surface. Left- and right-propagation spatiotemporal regions (4 mm × 1.75 ms size) were defined (*discontinuous-line boxes*). (**e**) Lamb wave phase speed estimation vs. frequency obtained from the two-dimensional Fourier analysis on each spatiotemporal propagating region in (**d**). Phase speeds at 2.5 kHz were extracted for each propagation side. (**f**) Lamb wave speed *c_L_*polar plot (in meters per second) was obtained by analyzing (**e**) in each corneal meridian. (**g**) Shear modulus (*G*) polar plot (in kPa) was calculated from (**f**) and (**c**) using the mRLFE model.^36^

The processing scheme applied to the OCT-based data along each corneal semi-meridian is presented in Figure 2 and follows these steps. (1) From the M-B scanning data, particle velocity (motion) videos (Fig. 2a) were reconstructed for each of the eight meridians, along with corresponding B-mode structural OCT images (Fig. 2b). Each motion video shows wave propagation toward the left and right direction as the excitation was performed in the corneal apex; therefore, we defined a left- and right-propagation spatial regions (4 mm × 4 mm size) in each B-mode image as shown green rectangular region of Figure 2b. (2) The average corneal thickness was computed in the left- and right-propagation spatial regions of each meridian by applying threshold-based segmentation to the B-mode images, enabling the generation of a polar map of the average corneal thickness (in micrometers) (Fig. 2c). (3) Space–time maps were constructed by tracking A0 Lamb waves from the motion videos along each meridian (Fig. 2d). Left- and right-propagation spatiotemporal regions (covering 4-mm in space and 1.75 ms in time) were defined (discontinuous line boxes in Fig. 2d). (4) A two-dimensional Fourier transform was applied separately to the left- and right-propagation spatiotemporal regions of the space–time map to estimate the frequency-dependent phase speed within each region^35^ (Fig. 2e). (5) The Lamb wave phase speed at 2500 Hz was extracted from the frequency-domain speed plot from both left- and right-propagation waves, and the resulting values were used to generate a polar plot of phase speed (in meters per second) across all eight meridians (Fig. 2f).

### Shear Modulus Estimation

The shear modulus was estimated using the modified Rayleigh–Lamb frequency equation (mRLFE) model,^36^^,^^37^ approximating the cornea as an isotropic, homogenous, and viscoelastic material. Although the cornea is inherently anisotropic and consists of layered microstructures,^9^^,^^38^ this first-order approximation facilitates direct comparisons of the biomechanical effects of various CXL treatments and protocols under consistent assumptions. For each cornea, the mRLFE mode was applied, using 16 paired measurements of Lamb wave phase speed and local corneal thickness obtained along 16 semimeridians. The model parameters included an excitation frequency of 2500 Hz, Poisson's ratio of 0.498 (assumed nearly incompressible), corneal tissue density of 988 kg/m^3^, aqueous humor density of 1000 kg/m^3^, and pressure-wave speed of 1500 m/s in cornea and aqueous humor. This analysis yielded 16 shear modulus estimates (in kilopascals), one for each semimeridian, visualized as a polar plot in Figure 2g.

### Statistical Analysis

Changes in corneal thickness and shear modulus, were analyzed using linear mixed-effects regression (LME)^39^ models, which account for the hierarchical and repeated-measures structure of the data. The models included fixed effects (protocol, photosensitizer, treatment phase, and angular position) and random effects (repeated measurements within each cornea) to capture subject-specific variability. This statistical framework enables robust inference based on analysis of variance (ANOVA) *P* values, and confidence intervals (CIs), providing an accurate and biologically consistent assessment of corneal biomechanical responses across experimental conditions.

The general LME model formulation is given by:

(1) $$\begin{array}{ccc} Y_{ijkl} & = & \mu +\beta_{P}\cdot P_{i}+\beta_{L}\cdot L_{j}\\ & & +\;\beta_{T}\cdot T_{k}+\beta_{A}\cdot A_{m}\\ & & +\;\beta_{PL}\cdot {\left(P\times L\right)}_{ij}+\beta_{PT}\cdot {\left(P\times T\right)}_{ik}\\ & & +\;\beta_{LT}\cdot {\left(L\times T\right)}_{jk}+\beta_{PLT}\cdot {\left(P\times L\times T\right)}_{ijk}\\ & & +\;\beta_{PA}\cdot {\left(P\times A\right)}_{im}+R_{l}+b_{l}P_{i}+\varepsilon_{ijkl} \end{array}$$

where *Y_ijkl_* is the response variable (shear modulus or thickness); μ is the overall intercept (grand mean); *P_i_* is the fixed effect of treatment phase (Virgin, Post-RB, or Post-CXL) with β_*P*_ as the estimate coefficient; *L_j_* is the fixed effect of protocol (DP, AC-1, or AC-2) with β_*L*_ as the estimate coefficient; *T_k_* is the fixed effect of RB photosensitizer (D, M, or TE) with β_*T*_ as the estimate coefficient; *A_m_* is the fixed effect of an angle (16 meridians for one subject); (*P* × *L*)_*ij*_ captures the interaction between treatment phase and protocol; (*P* × *T*)_*ik*_ captures the interaction between treatment phase and RB photosensitizer; (*L* × *T*)_*jk*_ captures the interaction between protocol and RB photosensitizer; (*P* × *A*)_*im*_ captures the interaction between treatment phase and angle; (*P* × *L* × *T*)_*ijk*_ captures the three-way interaction of treatment phase, protocol and RB photosensitizer; *R_l_* is the random intercept of the cornea, accounting for differences in baseline shear modulus and thickness across individual; *b_l_P_i_* is the random slope of treatment phase, allowing individual corneas to exhibit different responses in each treatment phase; and ε_*ijkl*_ is the residual error representing unexplained variability in the response variable that is not captured by the fixed or random effects (assumed to be normally distributed).

The LME model was fitted to the shear modulus shear modulus and corneal thickness data to generate predicted values (*Y_ijkl_*) as a function of the fixed effects: protocol, RB photosensitizer and treatment phase. For shear modulus and corneal thickness, 45 eyes × 3 measurement phases × 16 semimeridians = 2160 data points in each case were used for the LME fitting. To evaluate the performance of the LME model, we computed the marginal *R*^2^ (*R_m_*^2^) and conditional *R*^2^ (*R_c_*^2^) values. *R_m_*^2^ represents the proportion of variance of predictions (*Y_ijkl_*) explained by the fixed effects alone, and *R_c_*^2^ includes both fixed and random effects. The type III ANOVA test using Satterthwaite's method was used to assess the contribution of each fixed effect into shear modulus and thickness predictions.

### Effectiveness Factor (EF) for Corneal CXL

The EF was introduced to determine whether changes in shear modulus scale proportionally with corneal thickness:

(2) $$\begin{array}{c} EF_{\mathrm{Virgin}\;\to \;\mathrm{Post}-\mathrm{CXL}}=\frac{\Delta G_{\mathrm{Virgin}\;\to \;\mathrm{Post}-\mathrm{CXL}}}{\Delta TH_{\mathrm{Virgin}\;\to \;\mathrm{Post}-\mathrm{CXL}}}-1 \end{array}$$

where:

(3) $$\begin{array}{ccc} & & \Delta G_{\mathrm{Virgin}\;\to \;\mathrm{Post}-\mathrm{CXL}}\\ & & \;=\frac{G_{BL}+\left(G_{\mathrm{Post}-\mathrm{CXL}}-G_{\mathrm{Virgin}}\right)}{G_{BL}} \end{array}$$

quantifies the relative increase in corneal shear modulus (*G*) following CXL with respect to the baseline corneal shear modulus *G_BL_* calculated as the mean shear modulus of all corneas in the Virgin phase. Values of ΔG Virgin → Post - CXL  > 1 indicate a desirable increase in corneal stiffness, while ΔG Virgin → Post - CXL  < 1 represents a softening effect of the cornea. Additionally,

(4) $$\begin{array}{ccc} & & \Delta TH_{\mathrm{Virgin}\;\to \;\mathrm{Post}-\mathrm{CXL}}\\ & & \;=\frac{TH_{BL}+\left|TH_{\mathrm{Post}-\mathrm{CXL}}-TH_{\mathrm{Virgin}}\right|}{TH_{BL}} \end{array}$$

accounts for the relative undesired effect of the CXL, reflecting corneal thinning that could pose a risk of UV-damaging corneal endothelium or thickening due to corneal swealing, and this effect could be even more exacerbated by the ex vivo nature of the procedure. Such a change in corneal thickness was normalized with respect to the baseline corneal thickness (TH_*BL*_) calculated as the mean thickness of all corneas in the Virgin phase. ΔTH Virgin → Post - CXL  > 1 represents increase or decrease of corneal thickness after CXL.

Therefore, by evaluating Equation 1 using Equations 2 and 3, three scenarios can be distinguished: EF > 0 indicates that the increase in shear modulus exceeds the corresponding change in thickness (a desirable CXL effect); EF < 0 indicates that the increase in shear modulus (or possible decrease) is smaller than the thickness change (a undesirable trade-off among CXL effects); and EF = 0 denotes proportional changes in shear modulus and thickness (a neutral effect).

## Results

### Dresden Protocol

**Figure 3.**
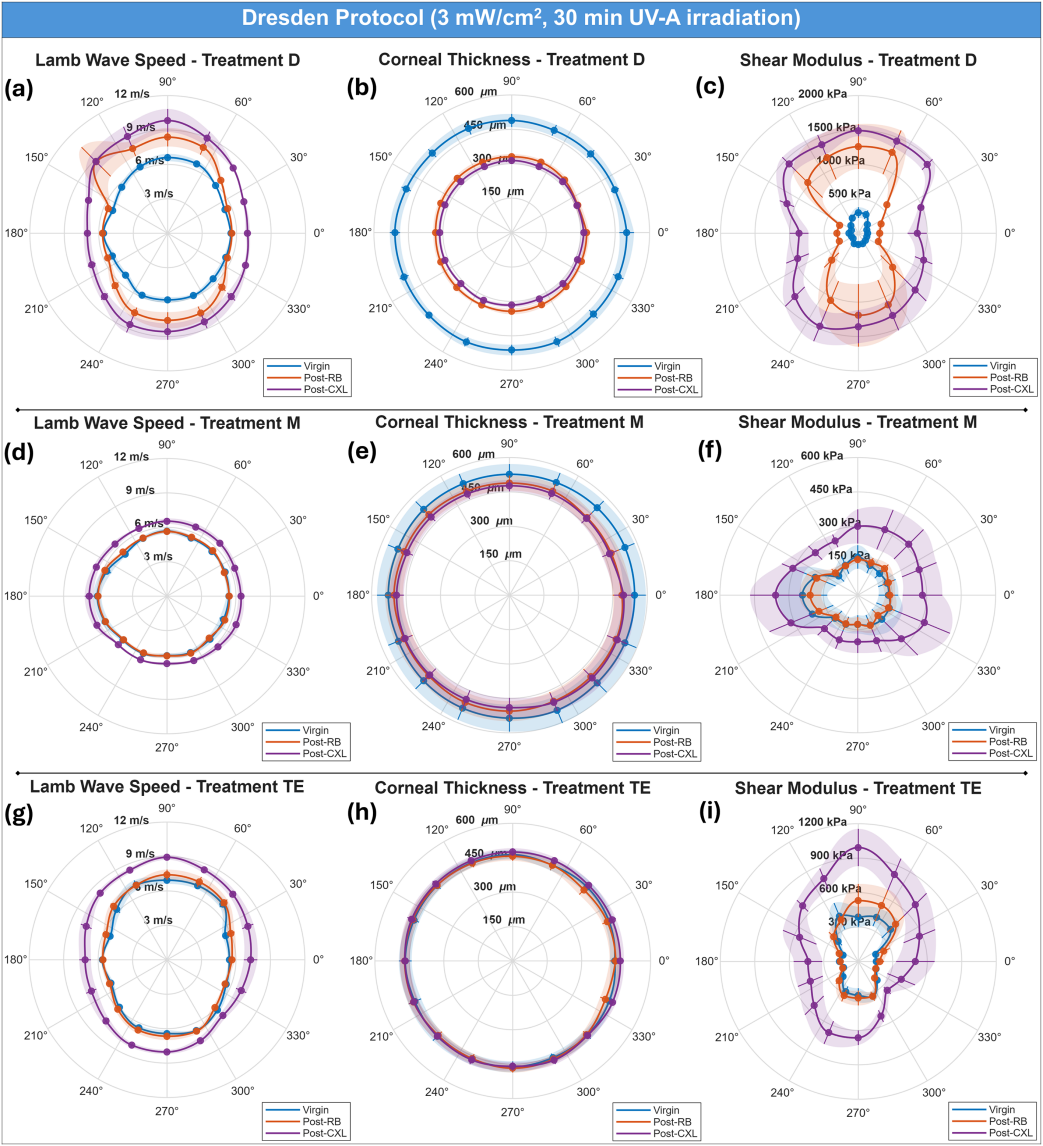
DP multimeridian estimations. Group average meridian-dependent polar plots of Lamb wave speed (*left*), average corneal thickness (*center*), and estimated shear modulus (*right*) are shown in corneas in the Virgin, Post-RB, and Post-CXL phases when using photosensitizers D (**a**, **b**, **c**), M (**d**, **e**, **f**), and TE (**g**, **h**, **i**) and DP (3 mW/cm^2^, 30 minutes of UV). Scattered points shown mean values within a group. Standard errors (SD) for each meridian are shown in the plots as shadow patterns and bars.

Figure 3 presents meridian-dependent polar plots of the average (across corneal samples) Lamb wave speed, corneal thickness, and shear modulus shear modulus of corneas in the Virgin, Post-RB, and Post-CXL phases of the DP when using photosensitizers D (Figs. 3a–c), M (Figs. 3d–f), and TE (Figs. 3g–i). Although the distribution of the polar plots across meridians is not necessarily uniform within single treatment phase, changes in Lamb wave speed, shear modulus (*G*) and average corneal thickness between phases are significantly uniform across meridians (*P* > 0.05), except for the cases of Lamb wave speed change (Post-RB–Virgin; *P* < 0.05), shear modulus change (Post-RB–Virgin; *P* < 0.01) and shear modulus change (post-CXL − Post-RB; *P* < 0.05) when using D photosensitizers.

**Figure 4.**
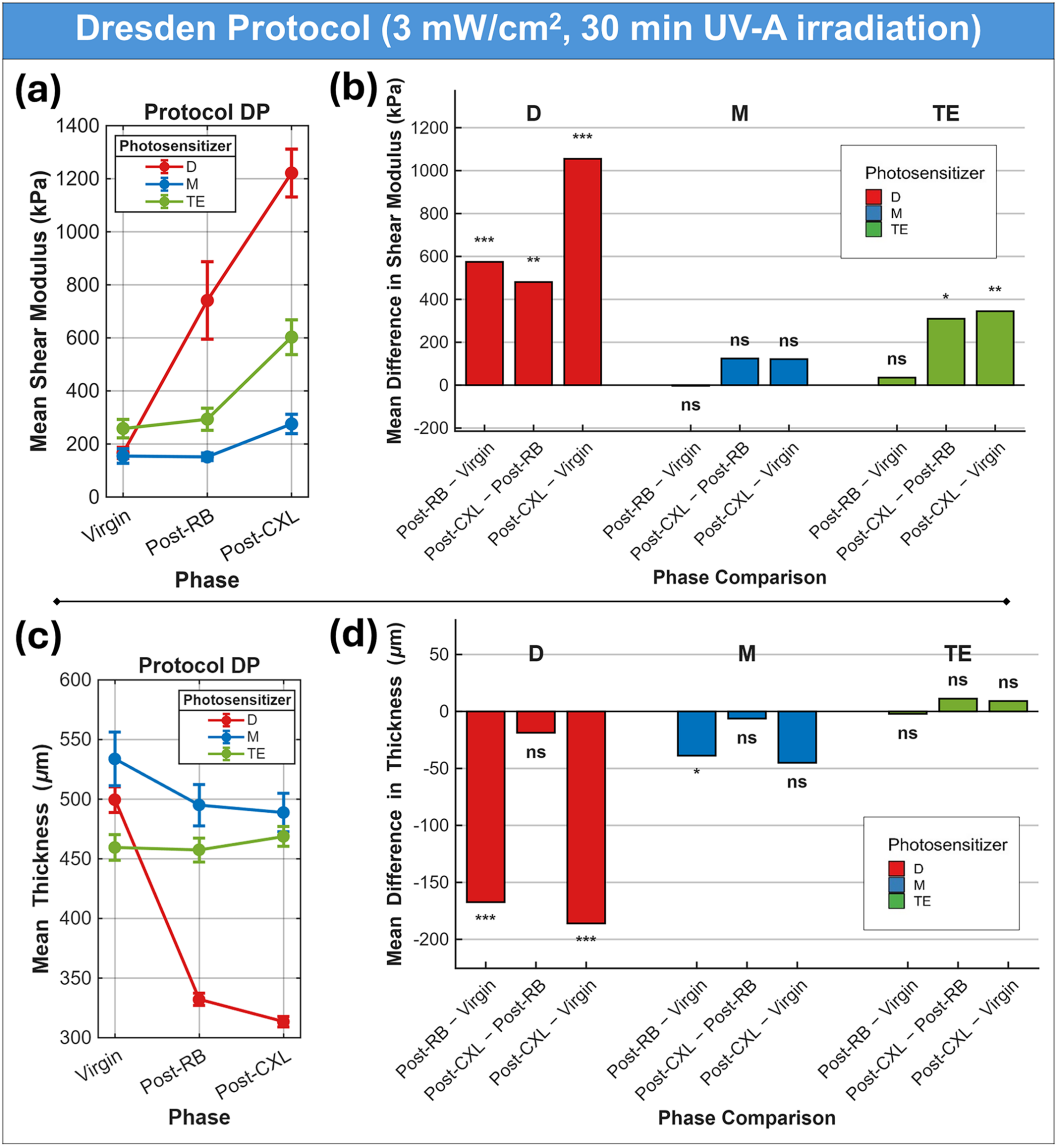
DP statistical results. (**a**) Estimated average shear modulus with 95% confidence limits vs. treatment phase for all RB photosensitizers. (**b**) LME-predicted mean difference of shear modulus across phases: Virgin → Post-RB, Post-RB → Post-CXL, and Virgin → Post-CXL for all RB photosensitizers. (**c**) Estimated average corneal thickness with 95% confidence limits vs. treatment phase for all RB photosensitizers. (**d**) LME-predicted mean difference of corneal thickness across phases: Virgin → Post-RB, Post-RB → Post-CXL, and Virgin → Post-CXL for all RB photosensitizers. Significant differences are presented as ***P < 0.001; **P < 0.01; and *P < 0.05. ns, not significant.

Figure 4a presents the mean values of shear modulus (*G*) across eyes for the three treatment phases and for each RB photosensitizer, with 95% confidence limits. The D photosensitizer shows the most pronounced significant increase of shear modulus during the Virgin–Post-CXL phase compared with M and TE. The shear modulus increased by 1054.9 kPa for D (*P* < 0.001), and 344.6 kPa for TE (*P* < 0.01), but no significant shear modulus change for the M photosensitizer, as shown in Figure 4b. Moreover, shear modulus increased significantly (574.4 kPa; *P* < 0.001) after RB soaking (i.e., the change from Virgin to Post-RB), but only for the D photosensitizer, highlighting the intrinsic stiffening effect of dextran-based RB solutions even in the absence of UV-A irradiation. Finally, the effect of UV-A irradiation on the increase in shear modulus (i.e., the change from Post-RB to Post-CXL) was significant for D photosensitizer (Δ*G* = 480.5 kPa; *P* < 0.001) and the TE photosensitizer (Δ*G* = 309.5 kPa; *P* < 0.05), while no significant change was observed for the M treatment.

Figure 4c shows the average tendency of corneal thickness across treatment phases for each RB photosensitizer. The D photosensitizer show the greatest thinning in corneal thickness from the Virgin to Post-CXL phase, with a reduction of 186 µm (*P* < 0.001), while M produced a significant change of 38.7 µm (*P* < 0.05) only in the Virgin to Post-RB phase, and no significant change in TE (Fig. 4d). Across all treatments, most thinning occurred during the Virgin–Post-RB phase. In every case, corneal thickness changes between phases along the corneal meridians were spatially uniform (*P* > 0.05), indicating that the CXL-induced thinning was uniform across the corneal surface.

### Accelerated Protocol 1

**Figure 5.**
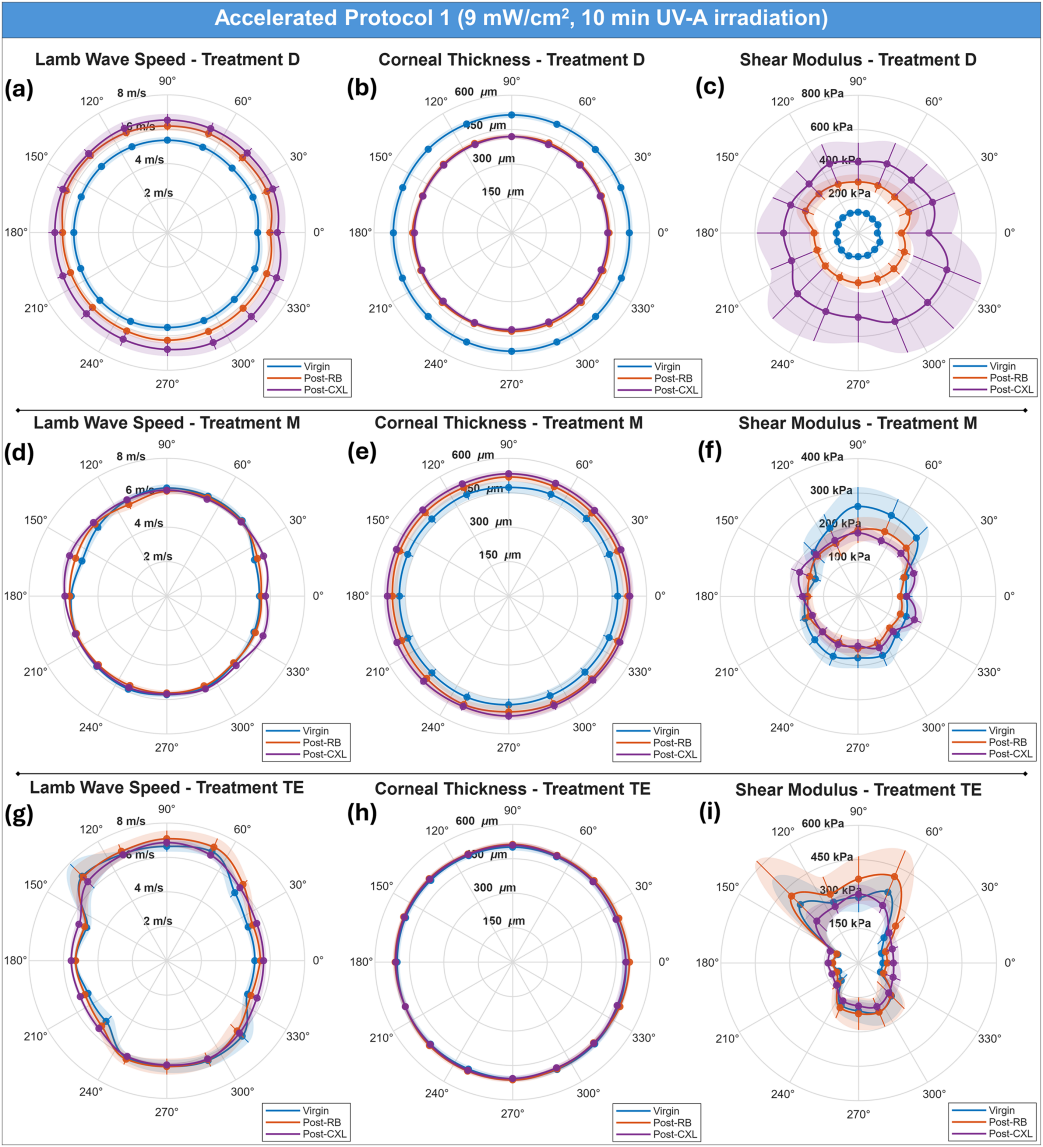
AP-1 multimeridian estimations. Group average meridian-dependent polar plots of Lamb wave speed (*left*), average corneal thickness (*center*), and estimated shear modulus (*right*) are shown in corneas in the Virgin, Post-RB, and Post-CXL phases when using photosensitizers D (**a**, **b**, **c**), M (**d**, **e**, **f**), and TE (**g**, **h**, **i**) and AP-1 (9 mW/cm^2^, 10 minutes of UVA). Scattered points shown mean values within a group. Standard errors (SD) for each meridian are shown in the plots as shadow patterns and bars.

Figure 5 presents meridian-dependent polar plots of the average (across corneal samples) Lamb wave speed, corneal thickness, and shear modulus shear modulus of corneas in the Virgin, Post-RB, and Post-CXL phases of the AP-1 when using photosensitizers D (Figs. 5a–c), M (Figs. 5d–f), and TE (Figs. 5g–i). Similar to the DP protocol, changes in Lamb wave speed, shear modulus (*G*), and average corneal thickness between phases are significantly uniform across meridians (*P* > 0.05), except for the case of Lamb wave speed change (Post-CXL−Virgin; *P* < 0.05), when using the M photosensitizer.

**Figure 6.**
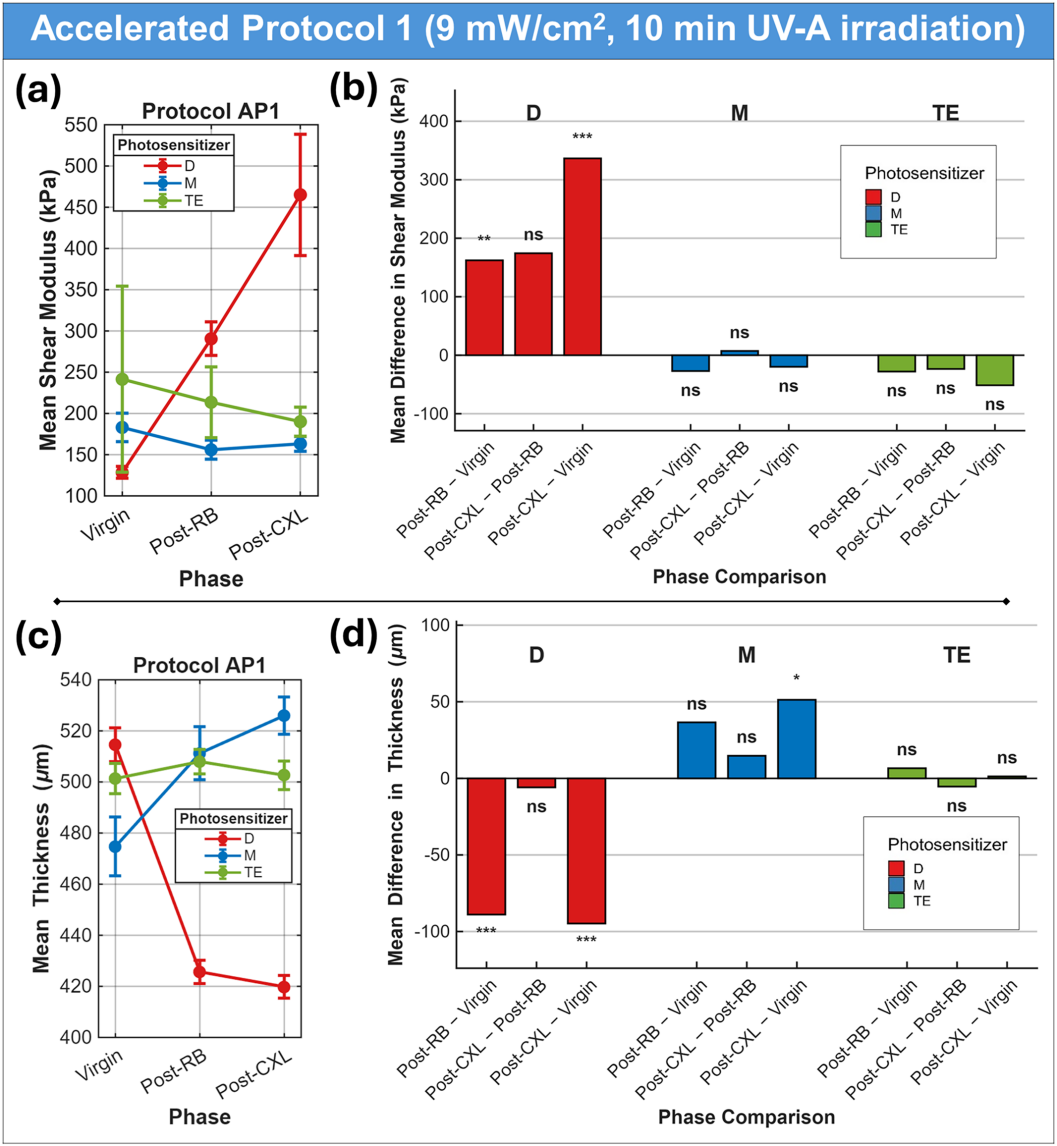
AP-1 statistical results. (**a**) Estimated average shear modulus with 95% confidence limits vs. treatment phase for all RB photosensitizers. (**b**) LME-predicted mean difference of shear modulus across phases: Virgin → Post-RB, Post-RB → Post-CXL, and Virgin → Post-CXL for all RB photosensitizers. (**c**) Estimated average corneal thickness with 95% confidence limits vs. treatment phase for all RB photosensitizers. (**d**) LME-predicted mean difference of corneal thickness across phases: Virgin → Post-RB, Post-RB → Post-CXL, and Virgin → Post-CXL for all RB photosensitizers. Significant differences are presented as ***P < 0.001; **P < 0.01; and *P < 0.05. ns, not significant.

Figure 6a presents the mean values of shear modulus (*G*) across corneas for the three treatment phases and for each RB photosensitizer, with 95% confidence limits. In the AP-1 protocol, only the D formulation produced a significant increase of shear modulus (336.4 kPa; *P* < 0.001) from the Virgin to the Post-CXL phase change, whereas M and TE showed no significant shear modulus change in any phase (Fig. 6b). Furthermore, with the D photosensitizer, the only significant increase in shear modulus (162.1 kPa; *P* < 0.01) occurred during the RB soaking phase (Virgin–Post-RB), with no additional stiffening effect after UV-A irradiation (Post-RB–Post-CXL).

Figure 6c shows the average tendency of corneal thickness across treatment phases for each RB photosensitizer. Significant corneal thinning was only observed in the D treatment, with reductions of 88.8 µm (*P* < 0.001) between the Virgin–Post-RB and of 94.7 µm (*P* < 0.001) between Virgin and Post-CXL. In contrast, the M treatment (epi-off) produced moderate corneal thickness increase of 51.2 µm (*P* < 0.05) between the Virgin and the Post-CXL phase, possibly due to the corneal swealing due to hydration during the experiments (Fig. 6d). No significant corneal thickness changes were observed for the TE photosensitizer in any treatment phase.

### Accelerated Protocol 2

**Figure 7.**
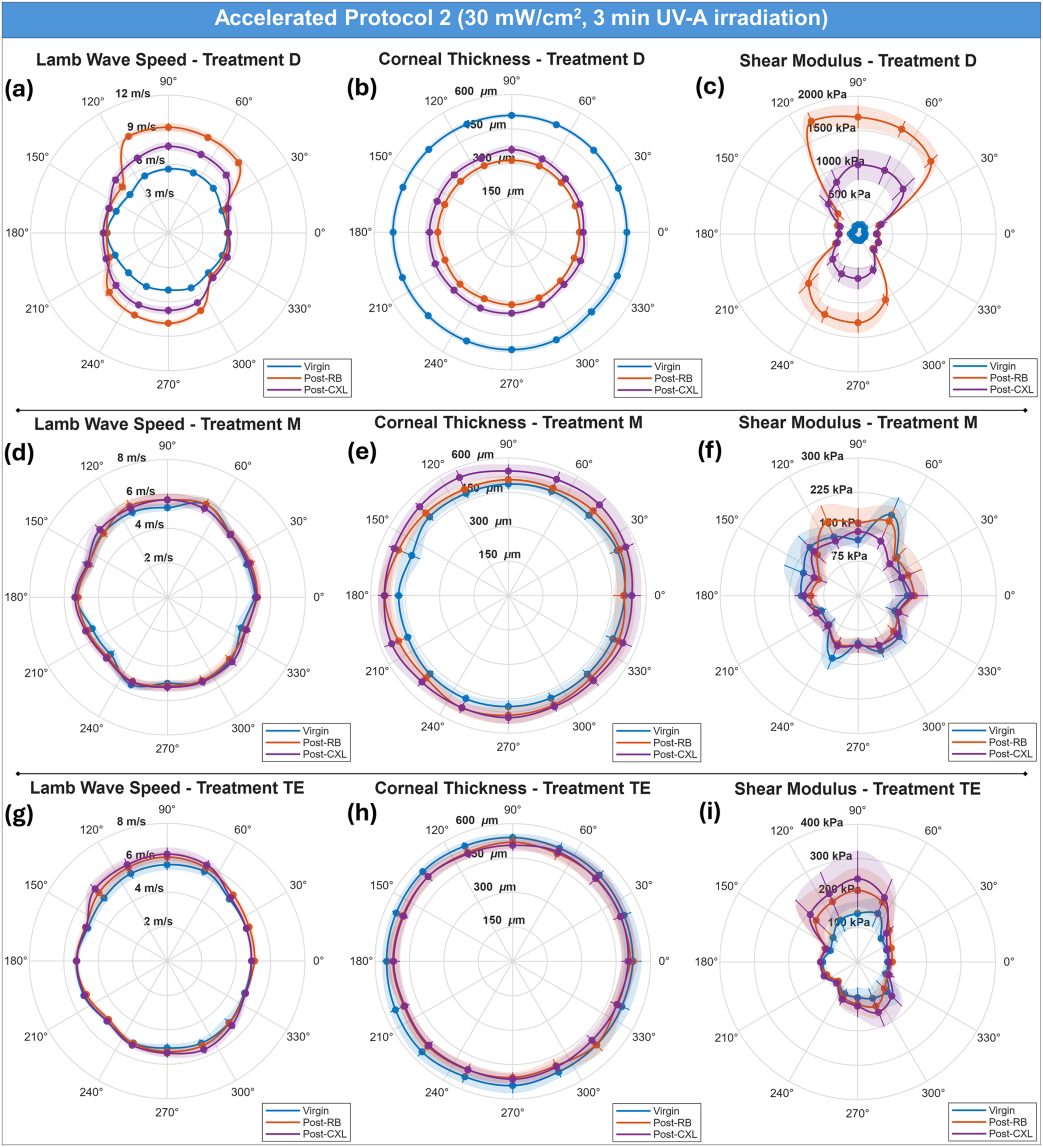
AP-2 multimeridian estimations. Group average meridian-dependent polar plots of Lamb wave speed (*left*), average corneal thickness (*center*), and estimated shear modulus (*right*) are shown in corneas in the Virgin, Post-RB, and Post-CXL phases when using photosensitizers D (**a, b, c**), M (**d, e, f**), and TE (**g, h, i**) and AP-2 (3 mW/cm^2^, 3 minutes of UVA). Scattered points shown mean values within a group. Standard errors (SD) for each meridian are shown in the plots as shadow patterns and bars.

Figure 7 presents meridian-dependent polar plots of the average (across corneal samples) Lamb wave speed, corneal thickness, and shear modulus of corneas in the Virgin, Post-RB, and Post-CXL phases of the AP-2 when using photosensitizers D (Figs. 7a–c), M (Figs. 7d–f), and TE (Figs. 7g–i). Changes in Lamb wave speed, shear modulus (*G*), and average corneal thickness between phases are significantly uniform across meridians (*P* > 0.05), except for the D case in Lamb wave speed and shear modulus changes along all phases (*P* < 0.001 for all cases). Figures 7a and 7c show a referable stiffening direction along 60° and 90°.

**Figure 8.**
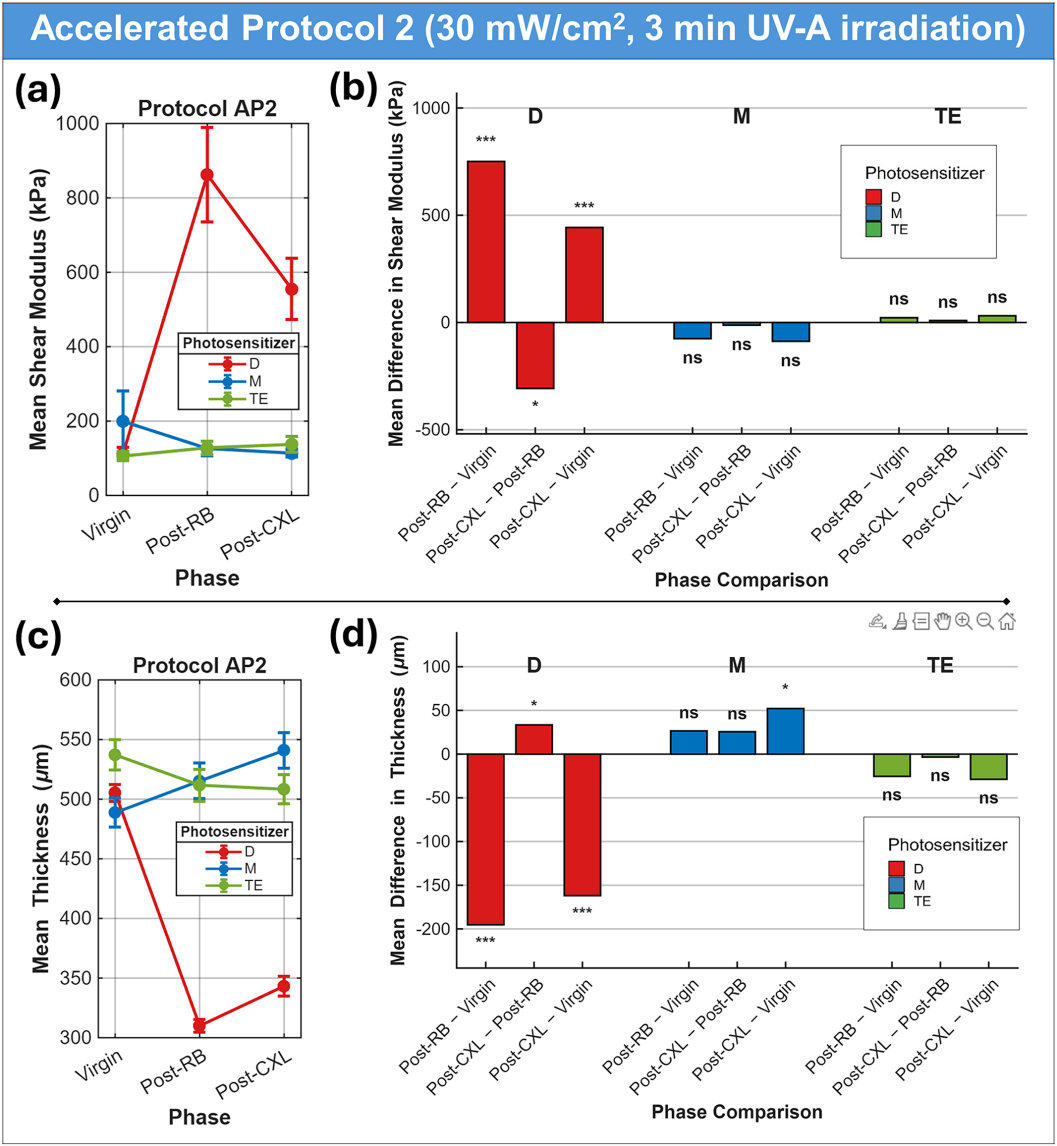
AP-2 statistical results. (**a**) Estimated average shear modulus with 95% confidence limits vs. treatment phase for all RB photosensitizers. (**b**) LME-predicted mean difference of shear modulus across phases: Virgin → Post-RB, Post-RB → Post-CXL, and Virgin → Post-CXL for all RB photosensitizers. (**c**) Estimated average corneal thickness with 95% confidence limits vs. treatment phase for all RB photosensitizers. (**d**) LME-predicted mean difference of corneal thickness across phases: Virgin → Post-RB, Post-RB → Post-CXL, and Virgin → Post-CXL for all RB photosensitizers. Significant differences are presented as ***P < 0.001; **P < 0.01; and *P < 0.05. ns, not significant.

Figure 8a shows the mean trends of shear modulus across treatment phases for each photosensitizer. In the AP-2 protocol, a significant increase in shear modulus (750.4 kPa; *P* < 0.001) was observed also during RB soaking (Virgin–Post-RB) with the D photosensitizer; no significant changes were detected for M and TE (Fig. 8b). Interestingly, for the D formulation, shear modulus decrease significantly during the irradiation phase (−307.4 kPa; *P* < 0.05), offsetting the previous gain and resulting in a net stiffening of 443 kPa (*P* < 0.001) by the end of the treatment (Virgin–Post-CXL). Figure 8c presents the trends in corneal thickness across treatment phases for each photosensitizer formulation. Significant corneal thinning was observed only with the D photosensitizer, showing reductions of 195.2 µm (*P* < 0.001) between the Virgin and the Post-RB phases, and 161.9 µm (*P* < 0.001) between the Virgin and Post-CXL phases. In contrast, the M formulation (epi-off) exhibited a moderate increase in thickness (52.1 µm; *P* < 0.05) from Virgin to Post-CXL, likely due to corneal hydration modulation during the experiments (see Fig. 8d). No significant corneal thickness changes were observed for the TE photosensitizer in any treatment phase.

### LME Regression Model

The LME model for shear modulus yielded a marginal *R*² ($R_{m}^{2}$) of **0.50** and a conditional *R*² ($R_{c}^{2}$) of **0.68**, indicating that fixed effects explain half of the variability in shear modulus, while subject-specific random effects provide a meaningful additional contribution. In contrast, corneal thickness showed a marginal *R*² of **0.60** and a markedly higher conditional *R*² of **0.95**, demonstrating that interindividual variability dominates thickness responses beyond the effects of experimental factors.

The ANOVA results show that, for shear modulus (see Table 3), all main effects and interactions are significant. The three most ranked effects are phase (*wp* = 46.80; *P* < 0.001); phase × photosensitizer (*F* = 46.45; *P* < 0.001); and photosensitizer (*F* = 40.63; *P* < 0.001). Angular position also has a strong effect (*F* = 19.73; *P* < 0.001), indicating that shear modulus varies significantly across different semimeridians of the cornea as shown by the nonuniform distributions in Figures 3c, 3f, and 3i; Figures 5c, 5f, and 5i; and Figures 7c, 7f, and 7i. All interaction terms are also significant, including phase × protocol (*F* = 11.60; *P* < 0.001), protocol × photosensitizer (*F* = 5.84; *P* < 0.001), and the three-way interaction (*F* = 6.31; *P* < 0.001). Notably, the phase × angle interaction is significant (*F* = 3.15; *P* < 0.001), indicating that some spatial pattern of shear modulus evolves across treatment phases as reported in the cases of Figures 3a and 3c; Figure 5d; and Figures 7a and 7c.

**Table 3.** Type III ANOVA (Satterthwaite Method) for Fixed Effects in the LME Model of Shear Modulus and Corneal Thickness

	Shear Modulus			Corneal Thickness		
Fixed Effect	*F*-Statistic	*P* Value	Ranking	*F*-Statistic	*P* Value	Ranking
Phase	46.81	<0.001	1	51.66	<0.001	2
Phase × photosensitizer	46.45	<0.001	2	55.17	<0.001	1
Photosensitizer	40.63	<0.001	3	23.25	<0.001	3
Angle	19.73	<0.001	4	3.95	<0.001	6
Protocol	13.98	<0.001	5	2.53	ns	
Phase × protocol	11.6	<0.001	6	8	<0.001	4
Phase × protocol × photosensitizer	6.31	<0.001	7	4.06	<0.001	5
Protocol × photosensitizer	5.84	<0.001	8	2.05	ns	
Phase × angle	3.15	<0.001	9	0.62	ns	

ns: non-significant.

In contrast, corneal thickness is primarily driven by phase × photosensitizer (*F* = 55.17; *P* < 0.001), phase (*F* = 51.66; *P* < 0.001), and photosensitizer (*F* = 23.25; *P* < 0.001), as shown in Table 3. Protocol alone is not significant (*F* = 2.52; *P* = 0.092); however, the significant phase × protocol interaction (*F* = 8.00; *P* < 0.001) shows that protocol-dependent differences emerge over the course of treatment. Although thickness may vary across angular positions (angular effect, *F* = 3.95; *P* < 0.001), the phase × angle interaction is not significant (*F* = 0.62; *P* = 0.947), indicating that this spatial pattern remains stable across treatment phases.

### Effectiveness Factor

The EF was computed using LME-predicted values for all protocol–photosensitizer combinations without restricting the analysis to statistically significant effects, to preserve comparability across conditions. Because the EF is derived from estimated changes in shear modulus and corneal thickness, its reliability depends on the precision of these underlying model estimates. Consequently, EF values associated with nonsignificant contrasts or wide CIs should be interpreted with caution, because they may reflect variability rather than robust biomechanical effects.

To quantify this uncertainty, CIs for EF were obtained via bootstrap resampling (5000 iterations) of the LME-predicted means for shear modulus and thickness. At each iteration, values were sampled from the estimated distributions defined by the model-based means and standard errors, and EF was recomputed using the formulation in Equations 2, 3, and 4. This approach accounts for uncertainty in both contributing variables and appropriately propagates it through the nonlinear EF formulation. Estimated marginal means were computed using equal weighting across angular positions, ensuring that each semimeridian contributed equally to the global corneal estimate.

**Figure 9.**
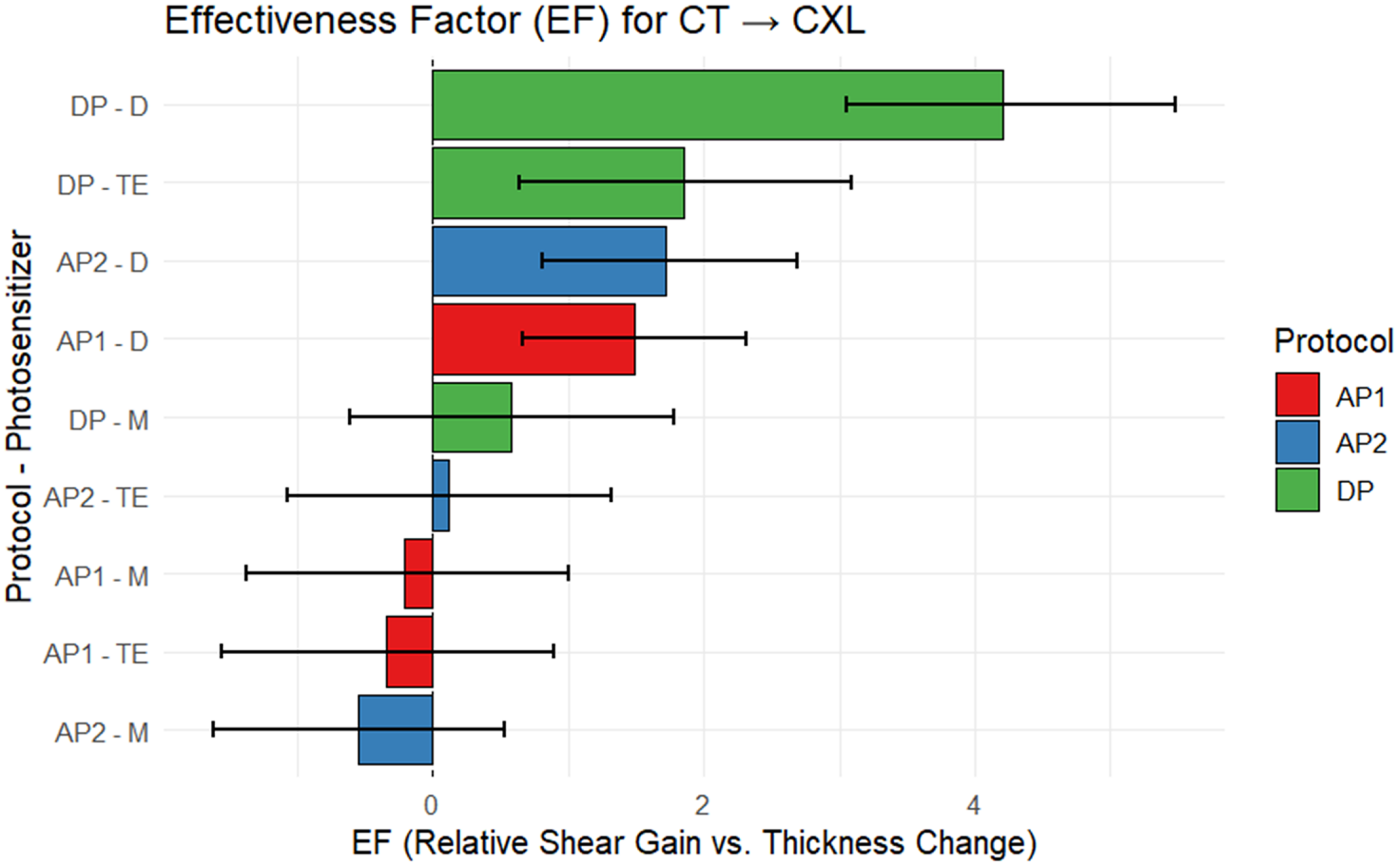
EF for all protocol–photosensitizer combinations. *Bars* represent EF values computed from LME-predicted means, and *error bars* indicate 95% CIs obtained via bootstrap resampling. Positive EF values indicate a greater relative increase in shear modulus compared with thickness change, whereas negative values indicate an unfavorable trade-off. CIs crossing zero denote effects that are not statistically distinguishable from a neutral balance between mechanical strengthening and structural change.

The EF values for all protocol–photosensitizer combinations are presented in Table 4 and illustrated in Figure 9. The DP–D combination exhibits the highest EF (EF = 4.21; 95% confidence interval [CI], 3.05–5.49), indicating a substantially greater relative increase in shear modulus compared with thickness change. This is followed by DP–TE (EF = 1.85; 95% CI, 0.64–3.09), AP-2–D (EF = 1.72; 95% CI, 0.80–2.69), and AP-1–D (EF = 1.48; 95% CI, 0.67–2.32), all demonstrating favorable biomechanical strengthening relative to structural alteration.

**Table 4.** EF Calculated for all Protocol Photosensitizer Cases (Equation 2) by Computing the Change of Shear Modulus (Δ*G*), and Change of Thickness (ΔTH) as Indicated in Equations 3 and 4, Respectively

Protocol-Photosensitizer	G_Virgin_, kPa	G_Post-CXL_, kPa	TH_Virgin_, µm	TH_Post-CXL_, µm	EF	Lower 95% CI of EF	Upper 95% CI of EF
DP-D	166.654	1221.613	499.537	313.534	4.208	3.051	5.485
DP-TE	258.124	602.765	459.528	468.655	1.847	0.642	3.088
AP-2-D	112.039	555.076	505.205	343.297	1.715	0.804	2.686
AP-1-D	128.485	464.956	514.582	419.836	1.483	0.665	2.317
DP-M	154.377	275.458	533.794	488.772	0.58	−0.618	1.78
AP-2-TE	105.94	137.353	537.165	508.375	0.114	−1.072	1.313
AP-1-M	182.987	163.186	474.7	525.987	−0.206	−1.381	0.998
AP-1-TE	241.495	190.13	501.361	502.648	−0.351	−1.557	0.895
AP-2-M	201.415	113.844	488.782	540.889	−0.555	−1.623	0.532

TH, thickness (µm).

Ranking reflects decreasing effectiveness.

Moderate effectiveness is observed for DP–M (EF = 0.58; 95% CI, −0.62 to 1.78) and AP-2–TE (EF = 0.11; 95% CI, −1.07 to 1.31), where CIs span zero, indicating that the balance between stiffness gain and thickness change is not statistically distinguishable from a neutral effect. In contrast, AP-1–M (EF = −0.21; 95% CI, −1.38 to 1.00), AP-1–TE (EF = −0.35; 95% CI, −1.56 to 0.90), and AP-2–M (EF = −0.56; 95% CI, −1.62 to 0.53) yield negative point estimates; however, their wide CIs also overlap zero, suggesting that these apparently unfavorable trade-offs are not robust.

Overall, these results indicate that the DP protocol, particularly when combined with D or TE photosensitizers, achieves the most favorable biomechanical efficiency. Importantly, the inclusion of bootstrap-derived CIs demonstrates that only a subset of combinations, primarily DP–D and, to a lesser extent, DP–TE and AP-2–D, show consistently positive and well-defined effects, while other combinations exhibit substantial uncertainty. This finding highlights the importance of accounting for variability when interpreting the EF, because point estimates alone may overstate the strength or direction of biomechanical effects.

## Discussion

In this study, we used ACUS-OCE for nondestructive, noncontact, and in situ biomechanical characterization of ex vivo rabbit corneas across distinct CXL phases. The ACUS-OCE enables rapid, multimeridian measurements of corneal wave propagation and corneal thickness along 16 angles spanning 360° around the corneal apex within a 2-second scan acquisition, allowing spatially resolved mapping at the Virgin, Post-RB (after 30 minutes of RB soaking), and Post-CXL (after 30, 9, or 3 minutes of UV-A irradiation and RB soaking) phases without interrupting the CXL procedure. Moreover, the ultrasonic excitation (500 kHz) provides consistent, repeatable stimulation of A0 Lamb waves (2.5 kHz) without inducing dehydration, tissue disruption, or macroscopic deformation, outperforming air-pulse OCE,^40^^–^^42^ contact-based elastography,^43^^–^^45^ uniaxial/biaxial extensiometry,^46^^,^^47^ and clinical tonometry, such as Corvis and Oculus Response Analyser.^48^^,^^49^

In wave-based OCE^50^ in the cornea, symmetric or antisymmetric zero-order wave modes are typically measured, and phase speed and thickness are used to estimate the shear modulus shear modulus via the mRLFE.^36^^,^^41^^,^^43^ This model allows for a robust estimation of the elastic part of shear modulus (and not the viscoelasticity) at a specific frequency, even in the absence of full phase speed dispersion measurements. Provided that the acoustic pressure field is incident normal to the corneal apex, the antisymmetric zero-order mode is selectively excited—as in this study—as demonstrated by Duvvuri et al.^41^ Because the mRLFE formulation incorporates both phase velocity and local thickness as independent variables, it inherently accounts for geometric variations, ensuring a consistent biomechanical interpretation of the shear modulus across different corneal thicknesses. Given the preferential lamellar organization of collagen fibers, the cornea is modeled as a transversely isotropic medium. In this configuration, the out-of-plane shear modulus—representing shear deformations in the *x*–*z* and *y*–*z* planes—differs from the in-plane shear modulus (µ or the *x*–*y* plane), which is more closely associated with tensile stiffness.^9^^,^^41^ Because the Lamb waves generated in this study primarily induce shear deformations perpendicular to the corneal surface, the calculated modulus corresponds to the out-of-plane shear modulus (*G*) as defined by Pitre et al.^9^

Our results demonstrate significant biomechanical effects of corneal CXL in rabbit corneas, and differences across different RB photosensitizers (D, M, and TE) and protocols (DP, AP-1, and AP-2), assessed at distinct treatment phases (Virgin, Post-RB, and Post-CXL). In all cases, changes in shear modulus were primarily driven by photosensitizer (*F* = 40.63) and, to a lesser extent, by protocol alone (*F* = 13.98), and protocol × photosensitizer (*F* = 5.84), indicating that the biomechanical response of the cornea is not solely dependent on individual factors, but is modulated by their specific combinations. Among all combinations, DP-D (Dresden protocol with dextran-based RB photosensitizer) produced the most significant corneal stiffening effect Post-CXL, with a mean increase in shear modulus of 1054.9 kPa (*P* < 0.001), but also induced the greatest corneal thinning, with a 186-µm decrease in thickness (*P* < 0.001). These findings are consistent with previously reported results using various elastography techniques.^5^^,^^8^^,^^10^^,^^12^

When comparing the impact of CXL photosensitizers only during the RB soaking phase (Virgin–Post-RB phase), we found that the D photosensitizer produced the most significant shear modulus increases and thickness decrease across all protocols (DP, AP-1, and AP-2), with no significant changes for M or TE. This finding highlights the biomechanical impact of dextran-based RB photosensitizers in the cornea in addition to the corneal thinning effect due to its high osmolarity that draws fluid out of the corneal stroma. Therefore, the dehydration caused by the D formulation could produce an increase of the shear modulus of the extracellular matrix of the stroma, associated with an increase in the propagation phase speed of A0 Lamb waves in cornea.^44^

We found the APs (AP-1 and AP-2) to be less effective in stiffening the cornea, compared with the standard DP, rendering some RB photosensitizer formulations ineffective when applied in an accelerated regime. For example, the epi-off M photosensitizer did not produce a significant increase in shear modulus, whereas epi-on TE only achieved significant stiffening if applied in the DP protocol. Similarly, the D photosensitizer achieved significant stiffening only with the AP-1 and AP-2 protocols after RB soaking (Post-RB). Interestingly, a decrease in shear modulus was observed during the irradiation phase (post-CXL) for the AP-2-D group. This reduction can be attributed to corneal swelling, which likely exerted a more dominant biomechanical effect than the CXL process itself during the 3-minute irradiation period. This is supported by the significant thickness increase reported for AP-2-D (see Figs. 8c, 8d) and aligns with previous findings by Singh et al.,^51^ who demonstrated that increased hydration levels lead to a measurable reduction in the corneal shear modulus. In this study, the APs exhibited a trend toward increased shear modulus, although these changes did not reach statistical significance. This lower stiffening efficacy compared with the standard DP is consistent with ex vivo observations using Brillouin microscopy,^16^ inflation test,^18^ stress-strain extensometry,^52^^,^^53^ and scanning acoustic microscopy^54^; and with clinical observations^14^^,^^15^^,^^17^ as reported in the literature. While comparing in vivo human data with ex vivo OCE measurements requires caution due to differences in hydration and IOP, both appear to reflect the same underlying biomechanical trends. The reduced efficiency of the APs may result from rapid oxygen consumption that outpaces stromal diffusion, leading to oxygen depletion and a shift from aerobic to a less efficient anaerobic CXL mechanism. In addition, shorter irradiation times limit RB diffusion and can accelerate RB photobleaching, thereby reducing the effective CXL depth.^21^ Furthermore, as Lamb wave speed represents a depth-averaged measurement of corneal stiffness, it may inherently underestimate the peak stiffness of the most superficial layers, which are primarily affected by the limited penetration of APs. To address this, stratified corneal models—similar to those used by Bekesi et al.^55^ to differentiate the penetration depths of Rose Bengal vs. RB CXL—could be applied to OCE data in the future. However, for the clinical purpose of preventing ectasia progression, the effective bulk stiffening provided by depth-averaged measurements may serve as a more robust predictor of overall structural stability than localized anterior stiffness alone.

When comparing the biomechanical effects of epi-on (TE) vs. epi-off (D, M) photosensitizers the DP-TE produced significant increase in shear modulus (*G* = 344.6 kPa; *P* < 0.01), demonstrating the potential of BKC-based transepithelial formulations to stiffen the cornea without inducing significant thinning or epithelial disruption. Similar behavior was previously reported by Armstrong et al.,^24^ who observed greater—but not statistically significant—stiffening using a BKC-EDTA epi-on RB formulation compared with the standard DP and other epi-on protocols, as measured using OCE. In contrast, no significant increase in shear modulus was detected for APs (AP-1, and AP-2) when combined with TE photosensitizer. Because the efficiency of the CXL procedure depends in part on the diffusion and penetration of RB, and this varies with protocols and photosensitizer formulation (epi-on and epi-off), depth-resolved methods of shear modulus should provide further mechanistic insights on CXL. Although in this study we report average shear modulus in each corneal meridians, possible depth-resolved reconstruction of shear modulus in cornea after CXL is being explored by Regnault et al.^56^ using wave-based OCE.

Corneal thinning was observed following full CXL (Virgin–Post-CXL) across all protocols (DP, AP-1, and AP-2) when using the D photosensitizer, consistent with known effects associated with 20% dextran content in the RB solution. In contrast, corneal thickening was observed in the AP-1 and AP-2 protocols when using the M photosensitizer. This response is likely due to endothelial cell disruption, which facilitates fluid influx and stromal swelling, as previously reported by other groups,^51^^,^^57^ although the extent of the differences of this effect in corneas in in vivo or ex vivo merits further investigation. Such swelling may contribute to corneal softening and could partially explain the reduced stiffening efficacy observed with the M photosensitizer in our measurements. Meanwhile, the TE photosensitizer did not induce significant changes in corneal thickness across any of the three protocols—DP, AP-1, and AP-2—underscoring the advantage of transepithelial (epi-on) treatments in maintaining structural corneal integrity. Importantly, it should be noted that corneal thinning induced by dextran-containing solutions is a transient effect during treatment that is typically not observed in clinical follow-up periods several weeks postoperatively.^22^ Within the context of our study, the proposed EF incorporates these transient thickness changes to provide a standardized metric for comparing biomechanical outcomes across different experimental groups. However, we acknowledge that the EF serves primarily as a comparative tool for the final state and does not fully account for how stromal hydration or compactness at the time of irradiation might fundamentally alter the photochemical efficiency of the CXL process itself. Although these ex vivo findings offer valuable insights into protocol-dependent stiffening, direct protocol optimization for clinical use would require further validation to account for the dynamic biological environment and oxygen-limited reactions present in vivo.

The baseline results of this study reveal that the rabbit cornea exhibits significant angular dependence of the shear modulus in the Virgin phase. This heterogeneity likely reflects the complex underlying lamellar organization and preferential collagen fiber orientation characteristic of the corneal stroma. However, most CXL protocol–photosensitizer combinations resulted in a uniform stiffening effect across all 16 measured semimeridians (*P* > 0.05), with exceptions observed when using D photosensitizer. Specifically, DP-D and AP-2-D led to nonuniform changes in shear modulus during both the RB soaking (*P* < 0.01) and UV irradiation (*P* < 0.05) phases. The observation that CXL may act as a global reinforcing agent to normalize inherent asymmetries remains preliminary. The specific mechanisms by which different photosensitizing agents interact with local stromal variations to either maintain or disrupt mechanical symmetry require further exploration in greater detail to fully understand the spatial impact of the treatment.

Some limitations in this study warrant consideration. Firstly, while ex vivo models may not fully replicate in vivo hydration dynamics, previous studies comparing CXL outcomes in both settings have shown that the general stiffening trends—particularly for the DP—remain consistent across both environments as shown in Bekesi et al.^58^ Consequently, despite the inherent differences in physiological conditions, our ex vivo findings provide a reliable perspective on the relative efficacy of various protocol-photosensitizer combinations. A second limitation is related to corneal dehydration. While balanced salt solution was applied via pipette to maintain corneal hydration between measurements, we anticipate minimal biomechanical softening compared with Phosphate-Buffered Saline (PBS) -based protocols.^51^ Unlike PBS, the divalent cations in balanced salt solution support the structural integrity of cellular tight junctions and metabolic pumps,^59^ which helps stabilize the stroma against the rapid swelling or breakdown often seen in ex vivo models. Future studies may benefit from humidity-controlled chambers to further standardize the hydration state across all experimental phases.^60^ A third limitation involves the inherent physics of Lamb wave propagation in the cornea. Lamb wave-based OCE methods provide an effective shear modulus shear modulus for the entire corneal thickness rather than a depth-resolved map, which may complicate the assessment of protocols that induce highly superficial stiffening (e.g., accelerated CXL). However, analytical modeling of bilayer media by Regnault et al.,^56^ consisting of a stiff layer over a soft layer demonstrates that Lamb wave speed is determined by the volume-weighted contribution of both layers. Specifically, a 3-fold increase in shear modulus constrained only to the anterior 10% of the cornea would still produce a statistically significant decrease in the measured wavenumber (k) and a corresponding increase in the phase velocity compared with untreated controls. Thus, while our current mRLFE model approximates the cornea as a homogeneous medium, it remains sensitive to the total structural reinforcement provided by thin crosslinked layers. Future work involving multilayered analytical models, as explored by Regnault et al.,^56^ will focus on reconstructing depth-dependent shear moduli to further refine the assessment of accelerated CXL protocols.

Other limitations of the study lie in structural assumptions of the cornea. Although all eyes were systematically aligned with the anatomical superior–inferior meridian, asymmetric patterns of shear modulus may arise from the nonuniform stress distributions inherent to corneal anatomy or from experimental artifacts introduced by the needles in the IOP control system. Although we minimized the needle gauge to reduce such effects, our analysis of shear modulus and corneal thickness per meridian was specifically designed to limit the impact of both physiological and artefactual asymmetries. Additionally, we noted inconsistencies in the Virgin to Post-RB corneal thickness variations within the same photosensitizer groups. For the dextran-based cases, although thinning was universal, the magnitude varied between batches in each protocol. The observed variability in corneal thinning after RB-dextran instillation is consistent with the wide range of osmotic effects reported in previous ex vivo literature. For instance, whereas De Paula et al.^61^ reported a 12.9% decrease in thickness, prior studies from our group using Scheimpflug^62^ and OCT^63^ imaging have observed thinning as high as 47% to 48%. This fluctuation likely stems from varying degrees of baseline edema in slaughterhouse-acquired samples, where more hydrated corneas may exhibit a nonlinear osmotic response to dextran. Similarly, although the M group generally exhibited the expected thickening associated with HPMC-based solutions (previously reported as a 9.1% increase),^61^ one case (DP-M) unexpectedly showed a decrease. Finally, the reported shear modulus values align with the expected order of magnitude for rabbit ocular tissue, which exhibits a fundamental discrepancy between in-plane tensile stiffness (measured in megapascals) and out-of-plane shear stiffness (measured in kilopascals) due to its transversely isotropic nature. As demonstrated by Villegas et al.^26^ using identical instrumentation, our results are consistent with previously reported rabbit data, and the higher magnitudes found in human studies such as that by Kirby et al.^8^ are attributable to species-specific differences in collagen density and tissue preparation.

In summary, this study provided in-depth comparative information of the biomechanical impact of UV-A RB-based CXL in ex vivo and in situ rabbit corneas (pressurized at 15 mm Hg) when using the DP and APs (AP-1 and AP-2), for different RB epi-off (D, M) and epi-on (TE) photosensitizers at three treatment phases (Virgin, Post-RB, and Post-CXL). We explored stiffening (increase of shear modulus) and corneal thinning effects at each phase for each protocol–photosensitizer combination. The proposed EF in Equation 2 and the EF results of Figure 9 shows that DP-TE offers a good compromise of both effects (EF = 1.847), while preserving corneal integrity and improving post-treatment recovery. Although the proposed EF metric quantifies the balance between corneal geometric changes and stiffening effects, it does not directly take into account corneal parameters such as hydration and compactness. Further analysis on how this EF is preserved over time and its capabilities of halting the progression of keratoconus is needed with in vivo clinical studies. The DP-D continues to have the strongest impact on the stiffening of corneas by the cost of corneal thinning, which makes it the most effective treatment (EF = 4.208). Unfortunately, according to our study findings, APs do not provide significant stiffening of the cornea, with the exception of AP-1-D, in which the stiffening is mostly produced in the RB-soaking phase and not during UV-A irradiation.

Future preclinical research can explore the effect of IOP on the shear modulus values, as well as the real-time biomechanical response of the cornea to CXL, by acquiring time-resolved OCE measurements throughout the course of different protocols and RB treatments to generate time-dependent stiffness profiles and investigate potential nonlinear effects related to UV-A irradiation dose and duration. Most important, the speed, safety, and adaptability of the ACUS-OCE technique enables the in vivo monitoring of corneal biomechanics both in animal models and in patients. In this context, future clinical studies can use ACUS-OCE to track the evolution of the EF throughout the postoperative recovery period and provide insight into the long-term biomechanical efficacy of various CXL strategies^10^^,^^30^^,^^31^ in keratoconus patients along many years, as shown in the study by Felter et al.^64^ The ability of ACUS-OCE to spatially resolve the corneal shear modulus in patients opens the possibility of selectively irradiating regions with differing stiffness, thereby enabling patient-specific customization of the CXL procedure and minimizing stiffness heterogeneity across the cornea.

## Conclusions

This study shows important biomechanical differences between combinations of RB photosensitizers (D, M, and TE) and protocols (DP, AP-1, and AP-2) during CXL of ex vivo rabbit corneas. We found that APs did not produce significant corneal stiffening for any RB photosensitizer, except by AP-1-D. Although the DP-D produces the strongest corneal stiffening effect, M treatment produces the lowest stiffening in all protocols attributed to absence of dextran. Interestingly, DP-D and DP-TE produce the greatest balance between shear modulus increase and corneal thickness decrease during ex vivo conditions, suggesting a possible research avenue for in vivo studies using DP-TE as a potential treatment with great compromise between biomechanical impact, corneal integrity, and faster postoperative recovery.
